# Mean-Field Effects on the Phosphorescence of Dinuclear Re(I) Complex Polymorphs

**DOI:** 10.1021/acs.cgd.1c01278

**Published:** 2021-12-17

**Authors:** Brunella Bardi, Anna Painelli, Monica Panigati, Pierluigi Mercandelli, Francesca Terenziani

**Affiliations:** † Department of Chemistry, Life Sciences and Environmental Sustainability, University of Parma, Parco Area delle Scienze 17/a, 43124 Parma, Italy; ‡ Dipartimento di Chimica, Università degli Studi di Milano, Via Golgi 19, 20133 Milano, Italy; § Consorzio INSTM, via G. Giusti 9, 50121 Firenze, Italy

## Abstract

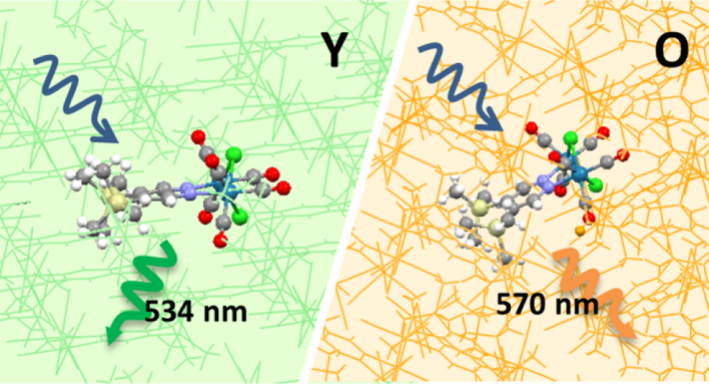

A computational study rationalizes the different phosphorescence colors of two highly emitting crystal polymorphs of a dinuclear Re(I) complex, [Re_2_(μ-Cl)_2_(CO)_6_(μ-4,5-(Me_3_Si)_2_pyridazine)]. The electrostatic interactions between the charge distributions on neighboring molecules inside the crystal are responsible for the different stabilization of the emitting triplet state because of the different molecular packing. These self-consistent effects play a major role in the phosphorescence of crystals made of polar and polarizable molecular units, offering a powerful handle to tune the luminescence wavelength in the solid state through supramolecular engineering.

## Abstract 2

The different phosphorescence colors of two polymorphs of a Re(I) complex are explained to be due to mean-field effects.

## Introduction

Optical properties of molecular assemblies and crystals are often rationalized invoking the so-called exciton approximation, which describes interacting molecules in terms of electrostatic interactions between their transition dipole moments.^1−10^ The strength of excitonic interactions depends on the magnitude of the transition dipoles, but also on their mutual orientation, so that a different arrangement of the monomeric units can explain different spectral properties, in an effect known as crystallochromism.^9,10^ The exciton model is well suited to rationalize recurrent features of aggregates, such as the splitting or shift of the absorption band with respect to the isolated (solvated) monomer, and the fluorescence quenching or amplification.^11^ Recently, the exciton model has been extended to account for intermolecular charge–transfer interactions, leading to fairly complex Hamiltonians as required to explain the appearance of additional bands not addressed in the standard exciton picture.^12−15^

In any case, for spin-forbidden singlet-to-triplet transitions, transition dipole moments vanish, so that the exciton picture would predict vanishing interactions as well. On these grounds, phosphorescence is expected to be unaffected by aggregation, ruling out the possibility of supramolecular engineering of phosphorescent materials.

The exciton model leads in general to an accurate description of optical spectra of aggregates and crystals of nonpolar and hardly polarizable chromophores,^3,4,16^ but a very different scenario occurs for polar and polarizable dyes.^13,17−20^ Indeed, the quite large permanent dipole moments of polar chromophores, either in the ground and/or in the excited states, strongly interact via electrostatic forces which are comparable with, if not larger than, their typical excitation energies, bringing about additional effects.

In the aggregate, a polarizable molecule readjusts its polarity in response to the local electrostatic field created by the neighbouring molecules in a feedback mechanism, a phenomenon known as mean-field effect.^18,19^ Mean-field effects set the basis for the nonadditive behavior observed in aggregates of polar and largely polarizable dyes, and add up to the effects due to excitonic coupling.^3,4,9,10,17,21−24^

Mean-field effects in aggregates of (multi)polar dyes have been extensively investigated with the help of theoretical models, and validated against experimental data. Their appropriate description was crucial to explain intriguing phenomena such as bistability^24^ and multielectron transfer,^20^ the amplification of nonlinear optical responses,^21,22^ and also to rationalize the unexpected fluorescence quenching of J-type aggregates of some quadrupolar dyes.^25,26^

Because mean-field effects arise from electrostatic interaction between the charge distributions of the dyes, they can be observed also in the absence of excitonic coupling. This opens an interesting scenario concerning phosphorescence. Indeed, these effects, which are also dependent on the supramolecular arrangement of the aggregate, could promote a stabilization/destabilization of the electronic states of the interacting dyes, shifting their emission energy and thus allowing to tune the phosphorescence spectral window through molecular packing.

**Figure 1:**
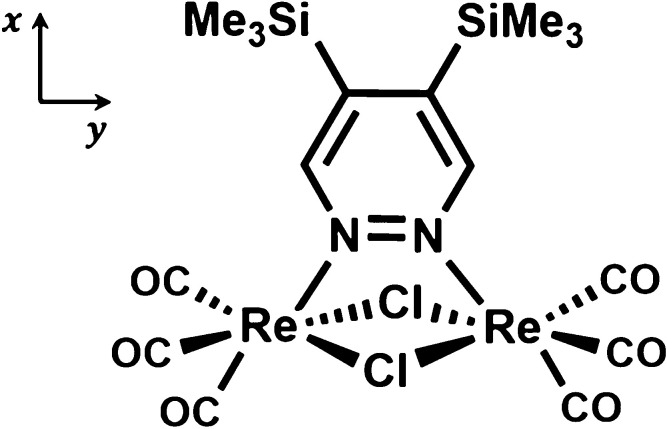
Molecular structure of dinuclear rhenium(I) complex **1**, [Re_2_(μ-Cl)_2_(CO)_6_(μ-4,5-(Me_3_Si)_2_pyridazine)].

In this context, dinuclear rhenium(I) complex **1** (Figure 1)^27,28^ provides an interesting case study. With two Re(I) atoms bridged by a pyridazine ligand, **1** is a member of the class of [Re_2_(μ-X)_2_(CO)_6_(μ-1,2-diazine)] (X = halogen) complexes, many of them displaying intense phosphorescent emission from triplet metal-to-ligand charge transfer (^3^MLCT) states.^29−31^ Members of this family have found applications as dopants of the emitting layer in OLED devices,^32^ as probes for cell imaging in biological applications,^33^ and as sensitizers for dye sensitized solar cells.^34^ The interest in **1** is motivated by the concurrence of different interesting properties, such as the high emission quantum yield (>0.5) in the solid state and the formation of two well-characterized concomitant polymorphs: **Y**, monoclinic, with half a molecule in the unit cell, and **O**, orthorhombic, with two molecules in the unit cell. The two polymorphs could be selectively obtained acting on the crystallization rate, and undergo a clean single-crystal-to-single-crystal transition at 443 K.^27^

Interestingly, the two polymorphs of **1** are characterized by markedly different spectroscopic signatures. Concerning absorption, the maximum of **O** is almost coincident with the absorption of the solvated monomer in a nonpolar solvent, while the maximum of **Y** is displaced to higher energy (by 0.2 eV). In emission, both polymorphs exhibit higher luminescence with respect to the solvated dye, a phenomenon known as aggregation induced emission^35−37^ and explained as a consequence of the restriction of the roto-vibrational motion of the SiMe_3_ groups occurring in the solid state.^27^ The phosphorescence spectrum of the two polymorphs is shifted to higher energy compared to the solvated molecule, but with different absolute shift, amounting to ∼0.15 and ∼0.3 eV for **O** and **Y**, respectively (with reference to toluene solution), which correspond to clearly distinguishable emission colors.

The absorption properties of the crystals were rationalized on the basis of the standard exciton model,^28^ thus excluding an effective role of intermolecular charge-transfer interactions. However, the different emission energy of **Y** and **O** claims for a different origin, since excitonic effects are negligible for forbidden transitions. Given the polar/polarizable nature of the complex, we envisage that mean-field effects may play an important role on phosphorescence of the crystals.

Herein, we report a detailed computational investigation of **1**, unravelling the role of mean-field effects on the luminescence color of its polymorphs. The first part of this work concerns the TDDFT description of the complex in solution, with emphasis on the nature of the triplet states involved in phosphorescence. Then, we will present a mean-field model for the molecules inside the crystal, toward the rationalization of experimental spectroscopic data. The study of **1** is not only significant in view of the peculiarity of the complex, but also highlights, with the help of computational tools, the importance of mean-field effects on the phosphorescence of polar and polarizable systems.

## Computational Details

All (TD)DFT calculations were performed with the Gaussian16 package.^38^ The hybrid functional M062X^39^ and the long-range corrected CAM-B3LYP functional^40^ were chosen for the calculations on the solvated dye. The Stuttgart–Dresden effective core potentials^41^ along with the corresponding basis set were adopted for the Re atoms, while the 6-31G(d,p) basis set was employed for the remaining elements. Stationary points located by the geometry optimizations were confirmed by frequency analysis. TDDFT calculations were run on the ground-state optimized geometry, at the same level of theory and with the same basis set, addressing the 15 lowest-energy electronic states. The solvent was introduced according to the polarizable continuum model (PCM) approach using either the linear response (LR) approach or a state specific approach (external iteration, EI).^42^ Transition energies were computed with linear response solvation and state-specific solvation.^43^ The Tamm–Dancoff approximation was imposed in TDDFT calculations on triplet states.^44^

To mimic mean-field effects in the crystal, we focused on model aggregates containing a limited number of molecules. TDDFT calculations were run in gas phase on one molecule in the aggregate surrounded by the equilibrium ESP charge distribution of the remaining molecules.^45,46^ The equilibrated ESP charge distribution was obtained by an iterative DFT calculation, starting from the ESP charges in gas phase as the initial guess. In each step, the charge distribution was updated with the newly calculated charges, and the cycle was repeated until the difference between the charges obtained in two subsequent steps was less than the predetermined threshold (<2% for all atoms). For the **O** polymorph, only one of the two molecules in the unit cell was considered at a time, replacing the other molecule with its charge distribution. The geometry of the molecules and the structure of the two polymorphs adopted in the calculations were extracted from crystallographic data.^27^ (TD)DFT calculations on the aggregates were performed with CAM-B3LYP functional and the same basis set as for calculations relevant to solutions.

## Results

### Modeling the Solvated Chromophore

Spectroscopic properties of **1** in solution can be found in literature.^27^ Absorption spectra show a broad and featureless band around 400 nm (∼3 eV), assigned to a ^1^MLCT transition. Absorption is sensitive to solvent polarity: the band maximum shifts to the blue as the solvent polarity increases (by ∼0.3 eV from toluene to acetonitrile), indicating that the ground state is more polar than the CT excited state. Compound **1** is weakly phosphorescent in solution (quantum yield < 0.06), with a broad spectrum in the orange spectral region and a monoexponential decay, with a microsecond lifetime.

TDDFT calculations on **1** were run in dichloromethane, a mildly polar solvent, and in acetonitrile, a highly polar solvent. Excitation energies of **1** in dichloromethane, computed with the CAM-B3LYP functional on the ground-state optimized geometry, are collected in Table 1.

**Table 1:** TDDFT Data of **1** in Dichloromethane Obtained with CAM-B3LYP Functional: Transition Energies and Wavelengths, Oscillator Strengths *f*, Components of the Transition Dipole Moment μ_*x*_, μ_*y*_ and μ_*z*_ (with Reference to the Cartesian Axes in Figure 1), and Main Character of the Transitions^a^

transition	energy (eV)	wavelength (nm)	*f*	μ_*x*_ (D)	μ_*y*_ (D)	μ_*z*_ (D)	type (>20%)
S_0_ → S_1_	3.62 (3.83)	342 (324)	0.000	0.003			H → L (94%)
S_0_ → S_2_	3.76 (4.00)	330 (310)	0.150		–3.243	–0.011	H – 1 → L (93%)
S_0_ → S_3_	3.79 (3.97)	327 (312)	0.006		–0.004	–0.644	H – 2 → L (94%)
S_0_ → S_4_	3.97 (4.25)	312 (292)	0.376	4.997			H – 3 → L (83%)
S_0_ → S_5_	3.99 (4.21)	310 (295)	0.003		–0.006	–0.475	H – 4 → L (59%), H → L + 2 (23%)
S_0_ → T_1_	3.43 (3.64)	361 (340)					H – 1 → L (73%)
S_0_ → T_2_	3.57 (3.78)	347 (328)					H → L (71%)
S_0_ → T_3_	3.57 (3.75)	347 (330)					H – 3 → L (60%)

a All quantities are obtained with LR-PCM, energies and wavelength in parenthesis refer to EI–PCM.

The lowest-energy transition, S_0_ → S_1_, is forbidden, while the two transitions contributing to absorption are S_0_ → S_2_ at λ = 330 nm and S_0_ → S_4_ at λ = 312 nm. While the estimated transition energies are somewhat overestimated, we ascribe the broad experimental absorption band observed at λ ≈ 384 nm to the contribution of both S_0_ → S_2_ and S_0_ → S_4_ transitions. The two transitions have different polarization: the S_0_ → S_2_ transition is polarized mainly along *y*, that is, the direction connecting the Re atoms (see Figure 1), the S_0_ → S_4_ is polarized along a perpendicular direction, specifically along the molecular *C*_2_ axis.

**Figure 2:**
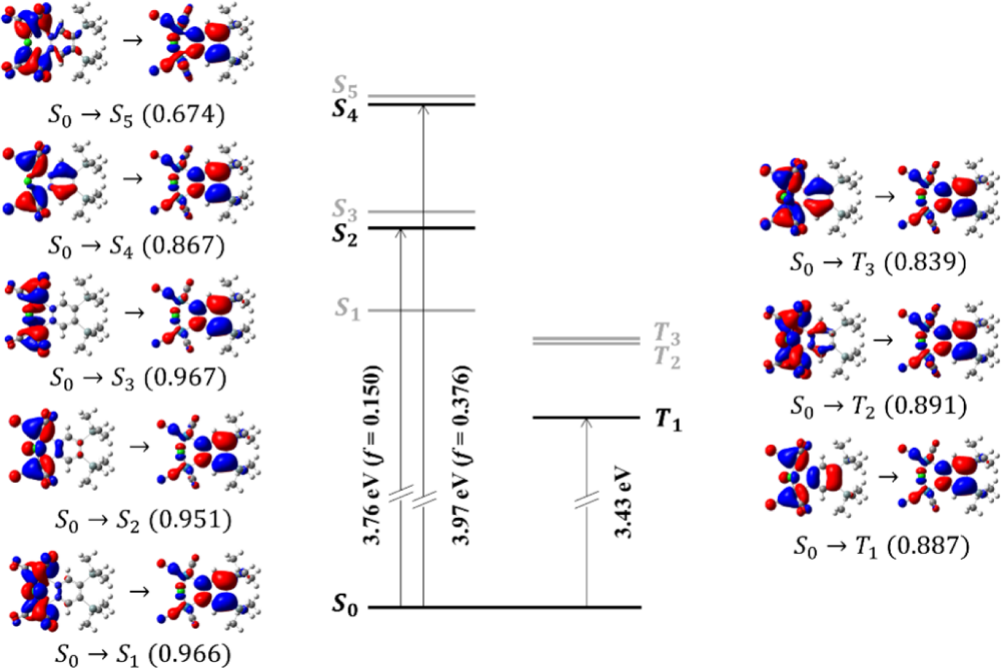
Sketch of the vertical electronic transitions of **1** and corresponding NTOs (isovalue 0.02) computed at CAM-B3LYP/6-31G(d,p) level in dichloromethane (energies are on scale). The states in bold are involved in absorption/phosphorescence; energies of the relevant transitions and oscillator strengths are reported on the arrows. Contribution of each NTO to the total excitations is given in brackets.

The inspection of the natural transition orbitals (NTOs)^47^ in Figure 2 suggests that the lowest-energy transitions of **1** have a clear CT character, and involve a charge migration from the region containing the metal centers and the ancillary ligands (CO and Cl^–^) to the pyridazine ring. Indeed, all excitations occur from one of the higher energy occupied orbitals (HOMOs) to the lowest unoccupied orbital (LUMO), which extends over the aromatic ligand. According to our calculations, **1** is highly polar in the ground state. Its permanent dipole moment, aligned with the *C*_2_ molecular axis, amounts to 17.05 D. The dipole moment decreases to 5.622 and 8.911 D after vertical excitation to S_2_ and S_4_, respectively, explaining the observed negative solvatochromic shift in absorption.

Results of the calculations in acetonitrile are provided in Table S1. Also, in this solvent, only two electronic transitions show sizeable oscillator strength, S_0_ → S_2_ and S_0_ → S_4_. As in dichloromethane, these transitions are polarized along mutually perpendicular directions and have a clear CT character (see NTOs in Figure S1). Compared to dichloromethane, the two transitions are blueshifted by ∼0.08 and 0.04 eV, respectively, (0.2 and 0.1 eV according to EI–PCM), comparing well with the experimental shift of ∼0.2 eV observed between these two solvents. TDDFT calculations in dichloromethane were also performed with M062X functional (Table S2 and Figure S2) obtaining similar results.

Phosphorescence emission is expected to occur from the lowest-energy triplet state, at its equilibrium geometry. According to the NTOs in Figure 2, the S_0_ → T_1_ transition has the same nature as the lowest optically allowed excitation, S_0_ → S_2_, while the brightest state in absorption, S_4_, has a similar nature as T_3_. To unambiguously identify the triplet state participating to phosphorescence, we relaxed the geometry of both states in solution. Even if the energy difference is moderate (<0.35 eV, Table S3), T_1_ lies below T_3_ at their respective equilibrium geometry, unambiguously identifying T_1_ as the phosphorescent state of **1**. This result was confirmed by calculations with M063X functional on the two triplet states possessing the same character of the optically allowed transitions in absorption (T_1_ and T_2_, Table S3).

The geometry of **1** at the T_1_ minimum is distorted with respect to the ground state geometry (Figure S3). While the equilibrium geometry of the ground state (S_0_) is planar, a sizeable deviation from planarity is observed in T_1_. For instance, while in S_0_, the Re atoms lie on the same plane as the pyridazine ring, they are tilted out of plane in T_1_. The pyridazine ring itself loses its planarity, affecting the relative positions of the Si atoms as well. These structural modifications can be appreciated considering the variations of representative dihedral angles reported in Table S4.

The S_0_ ← T_1_ transition in dichloromethane is predicted at 1.70 eV (731 nm) with the EI–PCM solvation method and CAM-B3LYP functional. This transition implies an increase of the permanent dipole moment from 9.27 D (at T_1_ equilibrium) to 15.04 D after de-excitation. NTOs relevant to phosphorescence are reported in Figure S4.

### Modeling of the Crystal Polymorphs

From the crystallographic point of view, the two polymorphs of **1** can be conveniently described as layered structures.^27,28^ In **Y**, the molecules have the same orientation inside each layer, defined by the plane containing the pyridazine rings, and different layers are stacked alternating the direction of the molecular dipoles. In **O**, the chromophores are organized in a zig-zag motif inside each layer, with a tilt angle of about 70° between the pyridazine and the layer’s plane, and the direction of the macroscopic polarity alternates every two layers. However, the inter-layer distance is small (∼5–6 Å) and of the same order of magnitude as the intralayer distance, so that the structure of both polymorphs must be considered as three-dimensional.

It follows that the full TDDFT treatment of the polymorphs of **1** is computationally unfeasible. However, a different and computationally affordable strategy can be pursued, which allows us to single out and quantify the contribution of mean-field effects on the spectroscopic properties of the two polymorphs. Specifically, we restrict explicit TDDFT calculations on just one molecule that however experiences the electrostatic field generated by the equilibrium charge distribution of the neighboring ones, as to mimic the environment felt by the molecule in the crystal. In this way, only electrostatic interactions are accounted for in the calculations.

**Figure 3:**
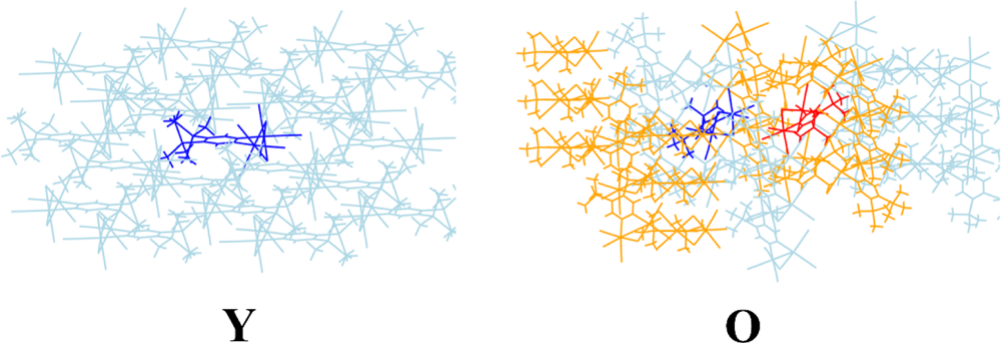
Two representative clusters for polymorphs **Y** and **O** discussed in the text, obtained surrounding the molecule(s) in the unit cell by its nearest neighbors for a total of 21 molecules for **Y** and 36 molecules for **O**. The molecules in dark blue/red are those explicitly considered in the TDDFT calculations, the remaining ones being replaced by their atomic charge distribution.

The electrostatic interactions decrease with distance, so that only a subset of molecules close to the target molecule are needed. To start with, we selected two representative clusters for **Y** and **O**, depicted in Figure 3, containing 21 and 36 molecules, respectively. These are the smallest structures accounting for nearest neighbor interactions. For polymorph **O**, the two molecules occupying different positions in the unit cell were separately considered in the calculation.

A self-consistent approach was followed to estimate the equilibrium charge distribution of the chromophore. ESP charges obtained for a molecule in gas phase and in the two representative clusters are resumed in Table 2. For discussion purposes, the chromophore is nominally partitioned in three regions: two regions (labeled “Re(CO)_3_Cl”) are constituted by a metal center and its ancillary ligands CO and Cl^–^ and one (labeled “(Me_3_Si)_2_pyridazine”) coincides with the pyridazine ligand. A charge separation occurs in the ground state, where the “Re(CO)_3_Cl” groups bear a similar negative charge, indicating that they act as electron withdrawing moieties with respect to the pyridazine ligand. The charge distribution changes moving from the isolated molecule in gas phase to the crystal, where the charge separation increases by 25% for both polymorphs. Moreover, the charge distribution of the non-equivalent molecules of **O** is different, reflecting their different environment.

**Table 2:** Cumulative ESP Atomic Charges on Selected Fragments of **1** in Gas Phase (Crystallographic Geometry) and Surrounded by the Charge Distribution of Nearest-Neighbors in the Two Representative Clusters for **Y** and **O** (21 Molecules for **Y** and 36 for **O**, as in Figure 3)^a^

	**Y**		**O**	
fragment	gas phase	crystal	gas phase	crystal
Re(CO)_3_Cl	–0.255	–0.319	–0.283/–0.246	–0.375/–0.272
Re(CO)_3_Cl	–0.256	–0.326	–0.261/–0.278	–0.231/–0.294
(Me_3_Si)_2_pyridazine	0.511	0.645	0.545/0.524	0.606/0.566

a The two values reported for the **O** polymorph were calculated for the two molecules in the unit cell. Calculations were performed at CAM-B3LYP/6-31G(d,p) level of theory.

**Figure 4:**
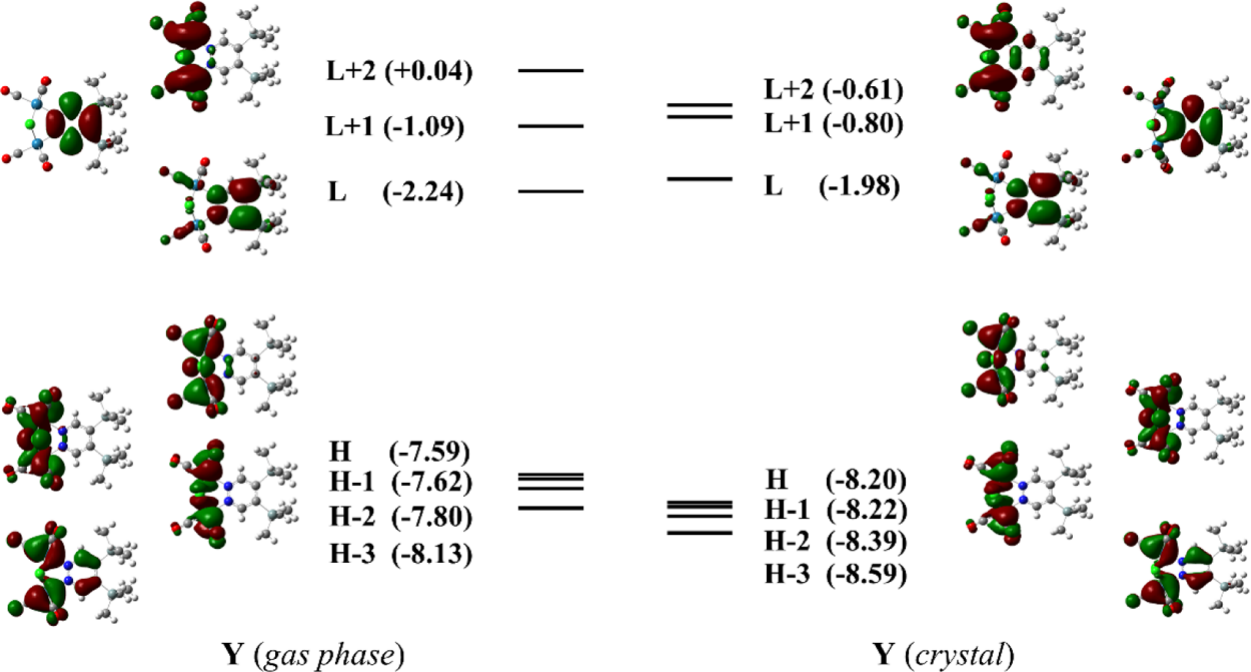
FMOs (isovalue 0.02) relevant to the **Y** polymorph. Left: FMOs of the monomer in gas phase; right: FMOs of a molecule surrounded by the charge distribution of 20 molecules of the crystal lattice. Orbital energies (in eV) are reported in brackets.

Frontier molecular orbitals (FMOs) of **1** calculated in gas phase and in a 21-molecule cluster (crystallographic geometry of **Y**) are shown in Figure 4 (FMOs relevant to polymorph **O** can be found in the Supporting Information, Figure S5). While occupied orbitals are localized on the Re atoms and the carbonyl ligands, LUMO and LUMO + 1 extend mainly over the region of pyridazine. Orbital shapes are basically unaffected by electrostatic interactions, and only a stabilization of the occupied orbitals is observed in the crystal.

To simulate phosphorescence spectra, TDDFT calculations were performed at the same level of theory. We will only address triplet transitions, where excitonic effects are negligible, while singlet transitions were already addressed in ref (28). TDDFT data relevant to the lowest-energy triplet transition are listed in Table 3 (TDDFT data on higher energy transitions can be found in Table S5). In the exciton picture, that is, neglecting mean-field contributions, identical transition energies are expected for the monomer and the two polymorphs because the transitions are spin-forbidden and hence have negligible transition dipole moments. However, in the crystal, the excitation energies relevant to the vertical S_0_ → T_*n*_ processes are shifted to higher energy compared to the gas-phase values, the magnitude of the shift depending on the crystal packing. Specifically, the vertical S_0_ → T_1_ excitation is blueshifted by ∼0.6 eV for the **Y** polymorph, and by only 0.2 eV for the **O** polymorph. For both polymorphs, the main contribution comes from the H → L process, with a minor contribution from H – 1 → L for polymorph **O**. Indeed, this transition implies charge migration from the region of the Re atoms toward the pyridazine ring, with a similar CT character as in solution.

**Table 3:** TDDFT Data on the S_0_ → T_1_ Transition of **1** in Gas Phase and Surrounded by the Charge Distribution of the Nearest–Neighbor Molecules as Depicted in Figure 3^a^

		**Y**		**O**		
	transition	energy (eV)	Type	energy (eV)	type	difference **Y–O**
gas phase (calc.)	S_0_ → T_1_	2.80	H → L (92%)	2.82/2.71	H – 1 → L (34/37%), H → L (56/54%)	
	S_0_ → T_1_^b^	1.97	H → L (89%)	1.97/1.97	H → L (89%)	
crystal (calc.)	S_0_ → T_1_	3.43	H → L (66%)	3.04/2.88	H – 1 → L (45/27%), H → L (41/59%)	
	S_0_ → T_1_^b^	2.20	H → L (81%)	1.97/2.03	H → L (90/89%)	0.20^c^
crystal (exp.)		2.32		2.17		0.15

a Calculations were performed in gas phase at CAM-B3LYP/6-31G(d,p) level of theory. The two values given for the **O** polymorph were obtained for the two molecules occupying non-equivalent positions in the unit cell.

b Calculated on the T_1_ equilibrium geometry.

c For the **O** polymorph, the average between the two values has been considered.

To compare with experimental phosphorescence energies, the geometry of the T_1_ state was relaxed both in gas phase and in the presence of the charge distribution of the surrounding molecules. The phosphorescence energy, obtained as the energy of the S_0_ → T_1_ process calculated at the T_1_ equilibrium geometry, amounts to 1.97 eV for the isolated molecule in gas phase, but is markedly affected by the electrostatic field of the cluster. Again, the magnitude of the spectral shift depends on the packing: emission of **Y** is blueshifted by ∼0.2 eV compared to gas phase, while emission of **O** is slightly blueshifted by ∼0.06 eV.

Noticeably, the difference between the calculated emission energies of the two polymorphs amounts to 0.2 eV, in good agreement with the experimental value (0.15 eV), confirming that the electrostatic effects experienced in the crystal, which are dependent upon the molecular packing, are responsible for the different emission colors of the two polymorphs of **1**.

To check for the effect of the dimension chosen for the sample aggregate on the predicted properties, we also included more molecules in the mean-field calculations. Aggregates containing up to 333 molecules for **Y** and 280 molecules for **O** were considered (Figure S6). ESP charges, summarized in Tables S6 and S7, are marginally affected by the size of the aggregate. As expected from the rapid decrease of electrostatic interactions over distance, the nearest-neighboring molecules are those giving the largest mean-field contributions. The phosphorescence energy of polymorph **Y** (Table S8) is independent of the dimension of the aggregate, while phosphorescence of **O** increases to ∼2.1 eV from 36 to 150 molecules while is not affected by a further increase of the aggregate’s size, giving an estimated Δ*E* ≈ 0.1 eV between the two polymorphs.

## Conclusions

Polar and polarizable molecules are highly sensitive to the environment. In a crystal (or aggregate), each molecule modifies its charge distribution in response to the electric field generated by the neighboring molecules in a feedback mechanism that can affect the spectroscopy of the ensemble. These effects, also called mean-field effects, are a fundamental component of the collective and cooperative behavior of polar and polarizable chromophores, in addition to the well-known excitonic coupling effects. Unlike excitonic effects, which are effective on absorption and fluorescence, electrostatic (mean-field) interactions are also of concern in phosphorescence, where the excitonic coupling, related to the interactions between transition dipole moments, is vanishing. These effects can be responsible for quite impressive modifications of the phosphorescence energy of crystals and aggregates, not only relative to the isolated (solvated) molecule, but also strongly dependent on molecular packing, leading to polymorphs with different emission colors.

Dinuclear Re(I) complex **1**, showing two highly phosphorescent crystal polymorphs with different emission colors, represents an instructive case study in this respect. (TD)DFT calculations run on a molecule experiencing, in a self-consistent way, the electrostatic field generated by the charge distribution on surrounding molecules accurately account for the different emission energies of the two polymorphs, pointing out its mean-field origin.

These findings are useful in a broader perspective. From the theoretical point of view, we demonstrated that the appropriate description of mean-field effects is crucial for the comprehensive understanding of cooperative effects in aggregates, in order to relate their supramolecular structure and their optical properties, demonstrating once again that the exciton model is not reliable for aggregates and crystals of polar and polarizable dyes.

Moreover, the observation of large effects of electrostatic intermolecular interactions on phosphorescence energies implies the possibility to exploit supramolecular and crystal engineering as a tool to tailor the emission properties of molecular materials made of polarizable dyes, and to obtain different luminescence colors from the same building blocks through the modification of their spatial arrangement. This strategy works not just in molecular crystals and films, but also in nanoparticles and multichromophoric systems. For this reason, CT phosphorescent dyes may attract further interest in the future, especially when combined with the possibility of switching between two or more (meta)stable phases through soft external perturbations, for example, pressure or heat.

## Supporting Information Available

TDDFT data for **1** in acetonitrile (CAM-B3LYP) and in dichloromethane (M062X); energies of the relaxed triplet states; comparison between ground and excited state geometry; NTOs for the S_0_ → T_1_ transition; FMOs for the **O** polymorph; TDDFT data of the lowest-energy triplet transitions of **Y** and **O**; and ESP atomic charges and emission energy of aggregates with a different number of molecules (PDF)
