# Supplementary material for: Mean-Field Effects on the Phosphorescence of Dinuclear Re(I) Complex Polymorphs
Source: Cryst Growth Des. 2021 Dec 17;22(1):772–8. doi: 10.1021/acs.cgd.1c01278 (PMC8765007; doi:10.1021/acs.cgd.1c01278)
Supplement: Supplementary file 1 — cg1c01278_si_001.pdf [file cg1c01278_si_001.pdf]

Mean-field effects on the phosphorescence of dinuclear Re(I) complex polymorphs

*Brunella Bardi,[a] Anna Painelli,[a] Monica Panigati,[b,c] Pierluigi Mercandelli,[b] Francesca Terenziani[a]**

[a] Department of Chemistry, Life Sciences and Environmental Sustainability, University of Parma, Parco Area delle Scienze 17/a, 43124 Parma, Italy.

[b] Dipartimento di Chimica, Università degli Studi di Milano, Via Golgi 19, 20133 Milano, Italy.

[c] Consorzio INSTM, via G. Giusti 9, 50121 Firenze, Italy.

* Email: francesca.terenziani@unipr.it

SUPPORTING INFORMATION

1. Solvated chromophore

Table S1. TDDFT data on **1** in acetonitrile obtained with CAM-B3LYP functional: transition energies and wavelengths, oscillator strengths f, components of the transition dipole moment μ_x, μ_x and μ_x (with reference to the Cartesian axes in Figure 1, main text), and main excitations. Energies in brackets were obtained with state-specific solvation.

Transition	Energy (eV)	Wavelength (nm)	f	μ_x (D)	μ_y (D)	μ_z (D)	Type (> 20%)
$S_0 \rightarrow S_1$	3.71 (4.04)	334 (306)	0.000	0.001	-	-	H → L (92%)
$S_0 \rightarrow S_2$	3.83 (4.21)	323 (294)	0.165	-	-3.369	-0.011	H-1 → L (90%)
$S_0 \rightarrow S_3$	3.87 (4.19)	320 (296)	0.009	-	-0.021	-0.781	H-2 → L (93%)
$S_0 \rightarrow S_4$	4.01 (4.37)	309 (284)	0.390	-5.059	-	-	H-2 → L (77%)
$S_0 \rightarrow S_5$	4.03 (4.27)	308 (290)	0.005	-	-0.003	-0.560	H-4 → L (46%)<br>H → L+2 (31%)

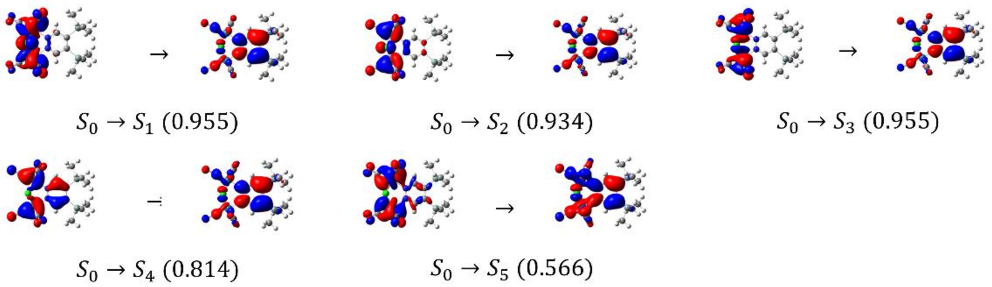

Figure S1. Natural transition orbitals (isovalue 0.02) for the five lowest electronic transitions of **1** obtained at TDDFT CAM-B3LYP/6-31G(d,p) level in acetonitrile. The weight of each NTO pair is given in brackets (only NTOs giving the main contribution are shown).

Table S2. TDDFT data on **1** in dichloromethane obtained with M062X functional: transition energies and wavelengths, oscillator strengths f, components of the transition dipole moment μ_x, μ_x and μ_x (with reference to the Cartesian axes in Figure 1, main text), and main excitations. Data on triplet transitions were calculated with TDA.

Transition	Energy (eV)	Wavelength (nm)	f	μ_x (D)	μ_y (D)	μ_z (D)	Type (> 20%)
$S_0 \rightarrow S_1$	3.93	315	0.000	0.003	-	-	H → L (87%)
$S_0 \rightarrow S_2$	4.02	308	0.124	-	-1.121	-0.002	H-1 → L (85%)
$S_0 \rightarrow S_3$	4.10	302	0.015	-	-0.015	-0.388	H-2 → L (83%)
$S_0 \rightarrow S_4$	4.17	297	0.000	-	0.006	-0.052	H-4 → L (39%)<br>H → L+2 (30%)
$S_0 \rightarrow S_5$	4.17	297	0.301	-1.715	-	-	H-3 → L (33%)<br>H-2 → L+2 (37%)
$S_0 \rightarrow T_1$	3.74	331	-	-	-	-	H-1 → L (64%)
$S_0 \rightarrow T_2$	3.80	326	-	-	-	-	H-3 → L (50%)<br>H-1 → L+2 (26%)
$S_0 \rightarrow T_3$	3.83	323	-	-	-	-	H → L (55%)

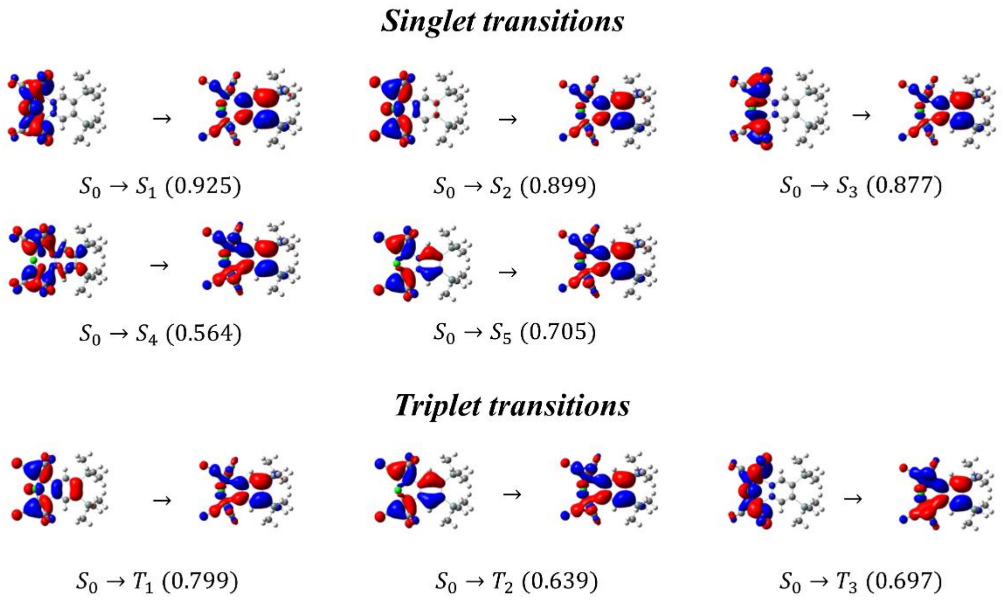

Figure S2. Natural transition orbitals (isovalue 0.02) for the lowest-energy electronic transitions of **1** in dichloromethane obtained at TDDFT M062X/6-31G(d,p) level. The weight of each NTO pair is given in brackets (only NTOs giving the main contribution are shown).

Table S3. Energies (in eV) of the relaxed triplet states calculated in dichloromethane with different functionals.

	State			
Functional	T_1	T_2	T_3	ΔE
CAM-B3LYP	2.85	-	3.17	0.32
M062X	3.11	3.47	-	0.36

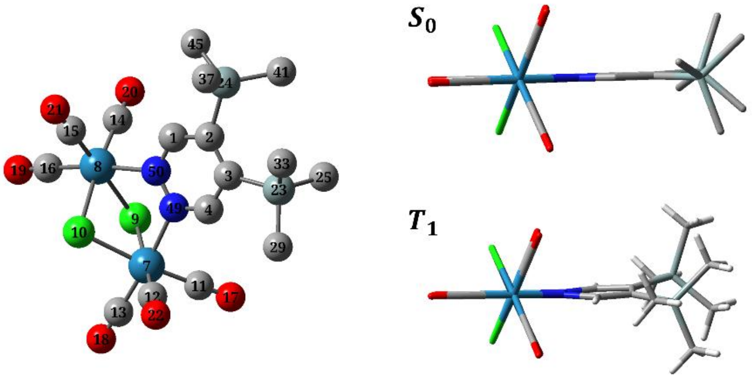

(a) **(b)**

Figure S3. Comparison between ground-state and excited-state geometry of **1**. (a) optimized ground state geometry with atom numbers (black: carbon, blue: nitrogen, red: oxygen, grey: silicon, green: chlorine, light blue: rhenium, hydrogen atoms are omitted for clarity); (b) side view of the molecule in the optimized ground state (S_0, top) and T_1 state (bottom). Geometries were optimized in dichloromethane.

Table S4. Values of selected dihedral angles (in degrees) of **1** in the ground-state (S_0) and in the T_1 state optimized geometry in dichloromethane (for atom numbering see the labels in Figure S3).

**Dihedral**	S_0	T_1
C13-Re7-Re8-C16	0.030	0.583
C1-C2-C3-C4	-0.227	-5.126
C1-N50-N49-C4	-0.008	-6.984
Si24-C2-C3-Si23	-3.865	-13.014

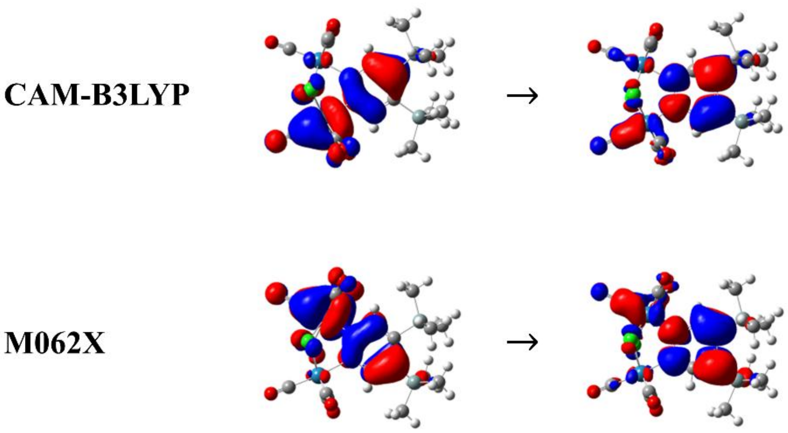

Figure S4. Natural transition orbitals for the $S_0 \rightarrow T_1$ transition at T_1 equilibrium geometry calculated with two different functionals in dichloromethane. The contribution of the NTO pairs to the total transition is 0.99.

2. Mean-field calculations

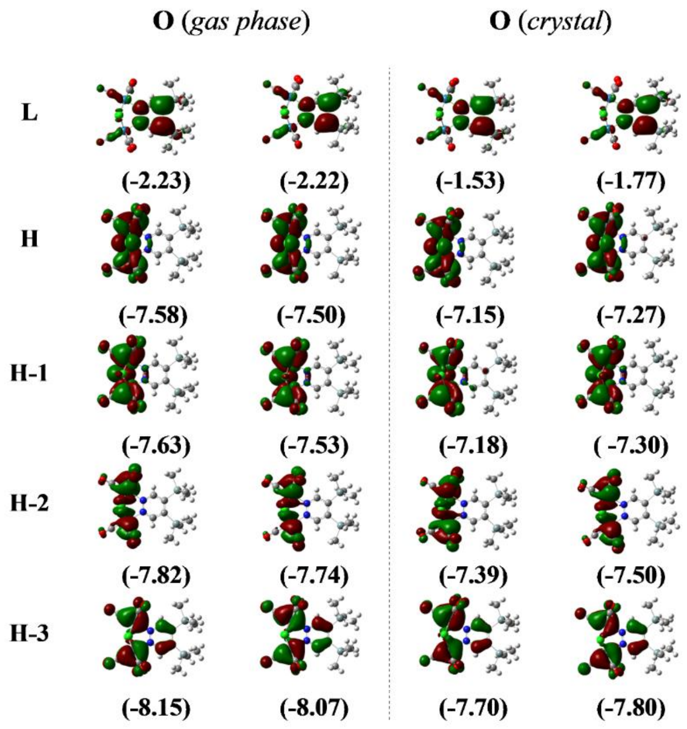

Figure S5. FMOs (isovalue 0.02) of **1** in gas phase (left) and surrounded by the charge distribution of 35 neighbouring molecules (right), both in the crystallographic geometry of **O**. The two columns refer to the two non-equivalent positions of the unit cell. The energies of the orbitals (in eV) are reported in brackets.

Table S5. TDDFT data on the vertical $S_0 \rightarrow T_n$ transitions calculated at CAM-B3LYP/6-31G(d,p) level for the monomer in gas phase and surrounded by the nearest-neighbours of the crystal. The two values for the **O** polymorph refer to the different molecules in the unit cell.

		Transition	Energy (eV)	Wavelength (nm)	Type (> 20%)
**Y**	Gas phase	$S_0 \rightarrow T_1$	2.80	442	H → L (92%)
		$S_0 \rightarrow T_2$	2.90	427	H-1 → L (97%)
		$S_0 \rightarrow T_3$	3.07	403	H-2 → L (97%)
	Crystal	$S_0 \rightarrow T_1$	3.43	362	H → L (66%)
		$S_0 \rightarrow T_2$	3.60	344	H-3 → L (73%)
		$S_0 \rightarrow T_3$	3.69	336	H-1 → L (85%)
**O**	Gas phase	$S_0 \rightarrow T_1$	2.82/2.71	440/458	H-1 → L (34/37%)<br>H → L (56/54%)
		$S_0 \rightarrow T_2$	2.89/2.78	429/446	H-1 → L (58/57%)<br>H → L (39/40%)
		$S_0 \rightarrow T_3$	3.08/2.99	403/415	H-3 → L (21%)<br>H-2 → L (74/80%)
	Crystal	$S_0 \rightarrow T_1$	3.04/2.88	408/431	H-1 → L (45/27%)<br>H → L (41/59%)
		$S_0 \rightarrow T_2$	3.14/2.99	394/414	H-1 → L (44/65%)<br>H → L (51/31%)
		$S_0 \rightarrow T_3$	3.30/3.17	375/391	H-3 → L (45/34%)<br>H-2 → L (45/57%)

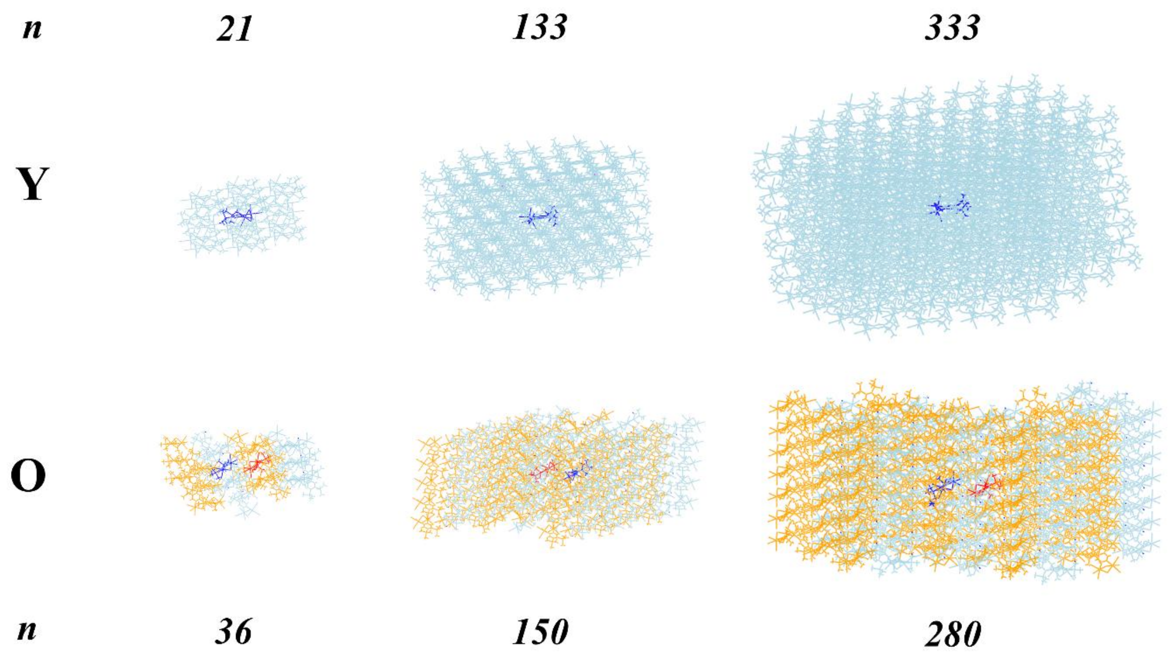

Figure S6. View of the clusters of different size (*n*: number of molecules) explored in this work. The molecule(s) in the middle of the cluster (in red for **Y**, in red and blue for **O**) were explicitly treated with TDDFT, the remaining molecules were replaced by their equilibrium charge distribution. For **O**, orange and light blue refer to molecules of different symmetry.

Table S6. ESP atomic charges on selected fragments of the chromophore (polymorph **Y**) calculated for aggregates containing different numbers of molecules.

	Number of molecules			
Fragment	1 (gas phase)	21	133	333
$Re(CO)_3Cl$	-0.255	-0.319	-0.310	-0.298
$Re(CO)_3Cl$	-0.256	-0.326	-0.317	-0.305
$(Me_3Si)_2$pyridazine	0.511	0.645	0.628	0.602

Table S7. ESP atomic charges on selected fragments of the chromophore (polymorph **O**) calculated for aggregates containing different number of molecules. The two values refer to the two non-equivalent monomers.

	Number of molecules			
Fragment	1 (gas phase)	36	150	280
$Re(CO)_3Cl$	-0.283/-0.246	-0.375/-0.272	-0.394/-0.251	-0.396/-0.250
$Re(CO)_3Cl$	-0.261/-0.278	-0.231/-0.294	-0.249/-0.325	-0.249/-0.327
$(Me_3Si)_2$pyridazine	0.545/0.524	0.606/0.566	0.643/0.576	0.645/0.577

Table S8. Energy of the $S_0 \leftarrow T_1$ transition calculated with TDDFT (CAM-B3LYP/6-31G(d,p)) on the relaxed (optimized) T_1 geometry in gas phase and surrounded by the charge distribution of *n* molecules.

**Y**		**O**	
n	Energy (eV)	*n*	Energy (eV)
1	1.97	1	1.97/1.97
21	2.20	36	1.97/2.03
133	2.21	150	2.10/2.14
333	2.20	280	2.11/2.15
exp	2.32	exp	2.17
